# Analysis and annotation of the hexaploid oat seed transcriptome

**DOI:** 10.1186/1471-2164-14-471

**Published:** 2013-07-11

**Authors:** Juan J Gutierrez-Gonzalez, Zheng Jin Tu, David F Garvin

**Affiliations:** 1 USDA-ARS Plant Science Research Unit and Department of Agronomy and Plant Genetics, University of Minnesota, St Paul, MN, 55108, USA; 2 Division of Biomedical Statistics and Informatics, Mayo Clinic, Rochester, MN, 55905, USA

**Keywords:** Transcriptome assembly, Oat, RNA-Seq, Tocol, Vitamin E, Avenanthramide, β-glucan, Trinity, Oases, Avena

## Abstract

**Background:**

Next generation sequencing provides new opportunities to explore transcriptomes. However, challenges remain for accurate differentiation of homoeoalleles and paralogs, particularly in polyploid organisms with no supporting genome sequence. In this study, RNA-Seq was employed to generate and characterize the first gene expression atlas for hexaploid oat.

**Results:**

The software packages Trinity and Oases were used to produce a transcript assembly from nearly 134 million 100-bp paired-end reads from developing oat seeds. Based on the quality-parameters employed, Oases assemblies were superior. The Oases *67*-kmer assembly, denoted *dn*OST (*de novo* Oat Seed Transcriptome), is over 55 million nucleotides in length and the average transcript length is 1,043 nucleotides. The 74.8× sequencing depth was adequate to differentiate a large proportion of putative homoeoalleles and paralogs. To assess the robustness of *dn*OST, we successfully identified gene transcripts associated with the biosynthetic pathways of three compounds with health-promoting properties (avenanthramides, tocols, β-glucans), and quantified their expression.

**Conclusions:**

To our knowledge, this study provides the first direct performance comparison between two major assemblers in a polyploid organism. The workflow we developed provides a useful guide for comparable analyses in other organisms. The transcript assembly developed here is a major advance. It expands the number of oat ESTs 3-fold, and constitutes the first comprehensive transcriptome study in oat. This resource will be a useful new tool both for analysis of genes relevant to nutritional enhancement of oat, and for improvement of this crop in general.

## Background

The genome and transcriptome of oats (*Avena sativa* L.) are one of the least explored among cereal grain crops. While the complexity associated with its large and repetitive genome (allohexaploid, 2n=6×=42) is an impediment, it is also clear that fewer efforts have been devoted to oat genome research. For instance, as of November 2012 there were only 28,938 oat nucleotide sequences in GenBank [1] which, assuming no sequence duplication, only represents approximately 0.1% of the estimated 13 Gb oat genome. This dearth of genome information is an obstacle to applying modern genetic and genomic methods for oat improvement, such as modifying the content and composition of various nutritional and health promoting compounds. Of particular interest are avenanthramides, tocols (vitamin E), and digestive fiber (β-glucans). The potential health benefits of avenanthramides in humans are largely based on their function as antioxidants [2]. Tocols, including vitamin E, prevent lipid oxidative damage [3-5]. A diet rich in the cell wall polysaccharide β-glucan is associated with a reduced risk of heart disease and reduced incidence of type II diabetes [6].

Despite interest in the health-promoting properties of oat, our understanding of the genetics and molecular properties of avenanthramide, tocol, and β-glucan content and composition is still in its infancy, in part due to the complexity and large size of the polyploid oat genome. Further, because oat has less global economic importance than other cool season cereal grains such as wheat and barley, less funding has been directed toward oat research, including the development of genomic resources. In the absence of a genome sequence and related genomic information, oat genetics and genomics can leverage genome information from related species. For instance, the genome sequence of the model grass *Brachypodium distachyon* (hereafter Brachypodium) [7] has shown potential for assisting oat genomics research because their genomes share large blocks of synteny despite differences in genome size and ploidy [8]. However, an ensemble of genomic resources for oat itself would be even more useful.

Recent advances in sequencing technologies, collectively known as next generation sequencing (NGS), have transformed genomic research. NGS has made possible high-throughput transcriptome sequencing (RNA-Seq), giving rise to a multitude of transcriptomes and transcript profiling studies in many organisms, including numerous plant species. For instance, RNA-Seq has provided evidence for protein-coding gene prediction and annotation [9-12], within-gene marker discovery [10,13,14], and accurate and sensitive gene expression measurement [15-18]. For species with a sequenced genome, RNA-Seq can assist in delimiting intron-exon boundaries and differential splicing to refine gene models [19]. In oat, cDNA sequencing has been employed for single nucleotide polymorphism (SNP) identification and marker development [20]. The authors outlined a rapid and effective high-throughput pipeline for SNP discovery and genotyping that could be used in other species with poorly-characterized genomes. Some of the SNPs developed were used to construct the first complete tetraploid oat linkage map [21].

Currently the most challenging aspect of RNA-Seq is the post-processing analysis of reads. A number of algorithms have been developed to accommodate analysis of massive amounts of RNA-Seq data. Typically, each algorithm is more appropriate for a specific type of sequencing technology, target sequence, organism, and experimental condition. Two of the software packages specifically developed for the assembly of transcriptomes are Velvet/Oases [22,23], collectively referred as Oases hereafter, and Trinity [24]. Both are able to assemble short reads without a reference genome by analyzing collections of *de Bruijn* graphs constructed based on series of overlapping *k*-mers. Velvet was initially designed for genomic DNA assembly, with Oases later added to address particulars of transcriptome assembly such as alternative splicing and high variability in gene expression that impact read coverage. Trinity was designed specifically for transcriptome assembly. Both packages are able to differentiate slightly dissimilar versions of a particular gene.

In this study, we employed high-throughput paired-end Illumina technology to generate 14.5 Gb of read sequence to explore the oat seed transcriptome. The specific objectives were i) to reconstruct a comprehensive transcriptome encompassing four stages of seed development; ii) to create, characterize, and annotate a gene expression atlas of the developing oat seed; and iii) to employ this atlas to examine gene expression associated with the synthesis of health-promoting compounds. This assembly constitutes the first comprehensive transcriptome study in oat seeds, and provides a valuable new resource for the oat community to assist efforts aimed at enhancing oat seed nutritional quality and other traits. In addition, it constitutes the first direct comparison between two of the most widely used assembly programs in a polyploid organism, and thus provides guidelines for future assemblies.

## Results

### Illumina sequencing and read assembly

**Figure 1:**
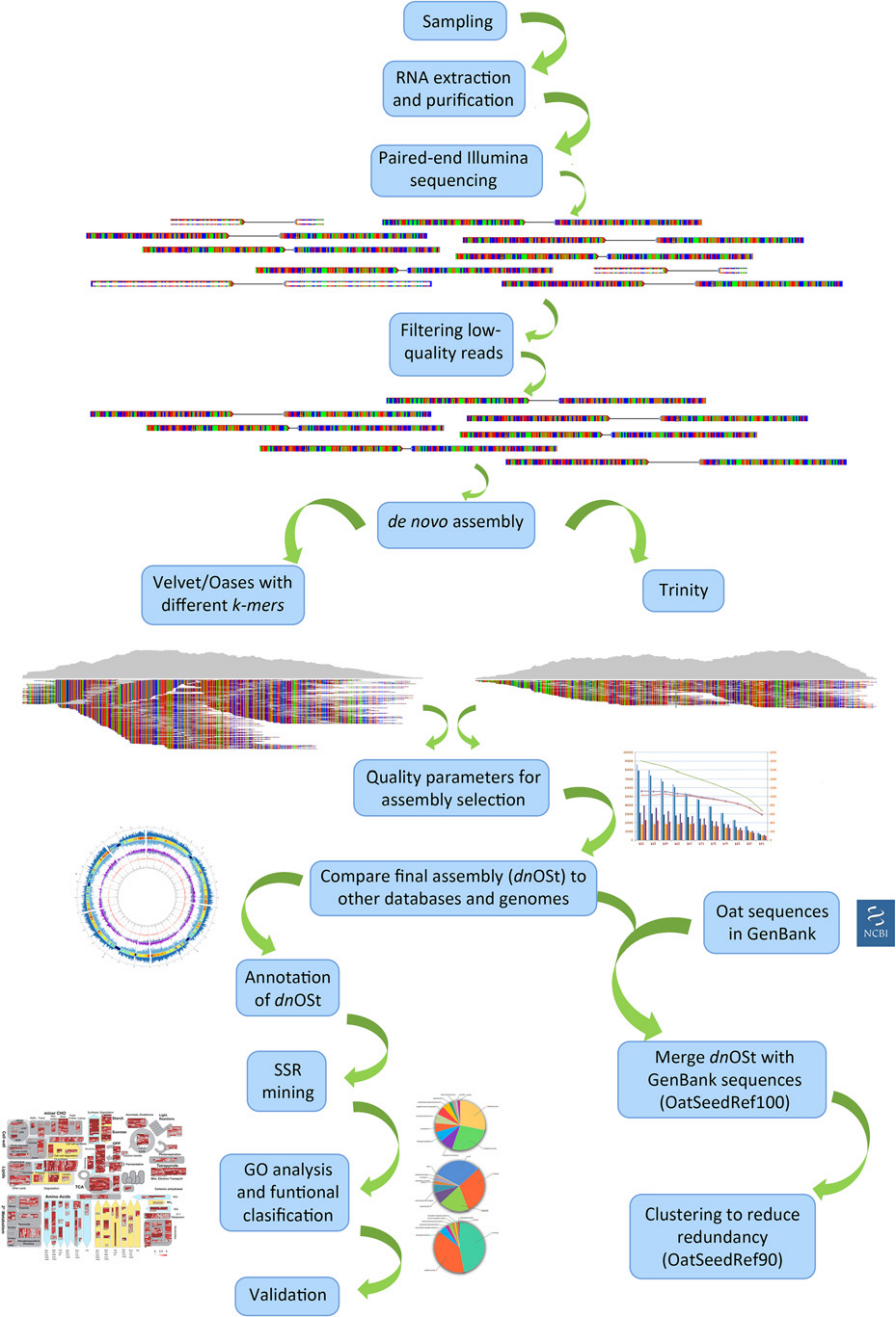
**Analysis flowchart.** Overview of the different steps followed for sequencing, assembly of the HiSeq2000 Illumina reads, and subsequent annotation and validation of the resulting transcriptome.

A flowchart overview of the steps followed in the assembly process is outlined in Figure 1. To obtain a broad sample of the oat seed transcriptome, four independent cDNA libraries were constructed from de-hulled oat seeds sampled at four developmental stages: 7, 14, 21, and 28 days after anthesis (daa). Libraries were sequenced using Illumina HiSeq 2000 technology, with nearly 145 million 100-base paired-end raw reads generated across the four stages. After removal of primer adaptor sequences and filtering low quality reads, 134 million high-quality reads remained. Erroneous base calls are more prone to happen near the 3′-end of the cDNA fragment. Thus, to increase the assembly accuracy, reads were further trimmed according to their individual base-call quality-score (QS) (See Methods for more details).

The 134 million reads were assembled with Oases and Trinity. Because Trinity demands considerably more computational resources, each of the four libraries had to be independently assembled when this package was used. In contrast the four libraries were combined within a single Oases assembly to increase the number of starting reads and the expected coverage. Additionally, while longer *k*-mers lead to more specificity (fewer spurious overlaps), they also lower coverage and sensitivity and thus perform poorly on genes expressed at low levels [23]. In assemblers where this parameter can be modified a balance must be reached during analysis. In its current version, Trinity does not allow *k*-mer value modification; rather it is fixed to a *25*-mer. However, with Oases several *k*-mer sizes (ranging 51-91nt) were tested. We could not test *25*-mer on Oases with the four libraries combined dataset due to memory limitations, since with shorter *k*-mers more *k*-mer-size fragments have to be allocated.

Here, we use the term ‘transcript isoform’ or ‘transcript’ to refer to each individual sequence in the assembly. The terms ‘*Locus’* or ‘*Loci’* are used to group together similar transcript isoforms. This assignment of isoforms to loci is performed by the Trinity and Oases assemblers based on sequence variations detected as the assembly process progress. Thus, separate transcript isoforms within a *Locus* might represent splice variants or other highly similar sequences such as homeoalleles (See Methods for more details).

A two-step evaluation was applied on all resultant Trinity and Oases assemblies to benchmark their quality. In the first step, several broadly used assembly-quality parameters were assessed. These included the number of assembled reads, the number of transcripts assembled, average transcript length, and the N50 value. Each parameter was calculated for the different *k*-mer Oases assemblies and for each of the four independently assembled libraries in Trinity. A summary of all quality parameters measured is shown in Additional file 1. An informative assay of assembly quality in the absence of a reference genome is to compare the assemblies against well-validated databases. Thus, in the second step both the Uniprot-Plants and UniRef50 databases, as well as the predicted complete set of translated coding sequences of Brachypodium, were selected to examine the quality of the assemblies (Additional file 2). For each *de novo* assembly tested, the following three types of transcript sequences representative of every *Locus* were compared with the sequences in the databases: i) the longest transcript isoform of each *Locus*; ii) the isoform with the highest confidence; and iii) non-redundant transcripts identified by clustering of all *de novo* transcripts. This clustering procedure reduces the size of the assembly by combining together highly similar isoforms (see Methods for details).

Based on the quality-parameters assessed, all Trinity assemblies were less accurate, had shorter transcripts, and contained fewer putative unique protein coding sequences than the assemblies constructed with Oases. Of the eleven *k*-mers tested in Oases, a good balance between transcript length, specificity, and diversity (number of transcripts) was found for the *67*-kmer assembly. This assembly, which contains 53,339 sequences, was termed *de novo* Oat Seed Transcriptome assembly (*dn*OST assembly) and used as the reference oat seed transcriptome assembly for further analysis (Additional file 3). Transcripts were further filtered to reduce presence of sequences with many undetermined calls (Ns), or shorter than 200 nt. The remaining 50,182 transcript sequences have been deposited at DDBJ/EMBL/GenBank under the accession GAJE00000000. The version described in this paper is the first version, GAJE01000000. To rule out the possibility that the superiority of Oases assemblies may be a consequence of the higher number of initial reads used, each library was also independently assembled with Oases. As expected, fewer transcripts were assembled in the individual libraries using the same *67*-mer: 13,228, 13,934, 10,104, and 16,854 for 7, 14, 21, and 28 daa libraries, respectively, as compared to *dn*OST (see below). Also, the average transcript length (879.5, 845, 777.9, and 753.8), and N50 (1,210, 1,143, 1,061, and 1,012) was shorter. However, these individual library Oases assemblies were superior to their counterpart library Trinity assemblies. In addition, the same *25*-mer that is fixed for Trinity was used with Oases on the individual libraries and produced 34,990 37,861, 31,651, and 44,330 transcripts of 738.4, 731.4, 672.3, and 791.0 average length, and a N50 of 1,328, 1,313, 1,198, and 1,377, for 7, 14, 21, and 28 daa libraries, respectively. All three parameters are higher than the ones obtained for Trinity assemblies.

### Analysis and annotation of *dn*OST

The *dn*OST yielded over 55 Mb of assembled sequence, with an average transcript length of 1,043 nt and average sequencing depth of 74.8×. Transcript length distribution is shown in Additional file 4. The *dn*OST assembly contains 53,339 transcript isoforms, which represent a total of 26,946 distinct assembled *Loci* (Table 1). As noted earlier, each *Locus* may include several highly similar transcript isoforms, whose sequence differences could reflect splice variants, homeologs and paralogs, and sequencing errors. For instance, when the longest transcript isoform per *Locus* was blasted (blastx) against the Uniprot-Plants database, there were 19,852 hits representing 73.7% of the 26,946 *Loci* (Additional file 2C). However, only 12,393 (46%) of them corresponded to unique Uniprot-Plants entries. Because only one isoform (the longest) per *Locus* was considered, the degree of redundancy may be attributable mostly to the inherent duplication of the hexaploid oat genome, and suggests that homeologous genes may be assembled within the same *Locus*, as well as in different *Loci*, depending on how divergent the homeologs are. In addition, to assist the identification of putative homeologous genes, regardless of the locus into they were assembled, a search was conducted with the *dn*OST assembly against itself (blastn, 1E-40, high-scoring pair identity [HSP]-id > 95%). Although families of close paralogs are most certainly present, due to the stricter parameters used, this search is more likely to retrieve homeologous relationships by grouping together highly similar transcript isoforms. We found 22,818 relationships and denoted the results the Homeologous Set File (HSF). Within the HSF, the longest transcript was chosen as the representative of the *Locus* (Additional file 5).

**Table 1:** ***k-67 *assembly ( *dn *OST) statistics**

Total number of reads (filtered)	133,963,046
Total sequence of reads (nt)	12,848,804,660
Assembled reads	43,330,506
Transcript isoforms	53,339
Num of *Loci*	26,946
Total transcriptome length (nt)	55,645,028
Ave transcript isoform length (nt)	1,043
Min transcript isoform length (nt)	100
Max transcript isoform length (nt)	12,827
Ave coverage (×)	74.8

nt: nucleotides.

**Figure 2:**
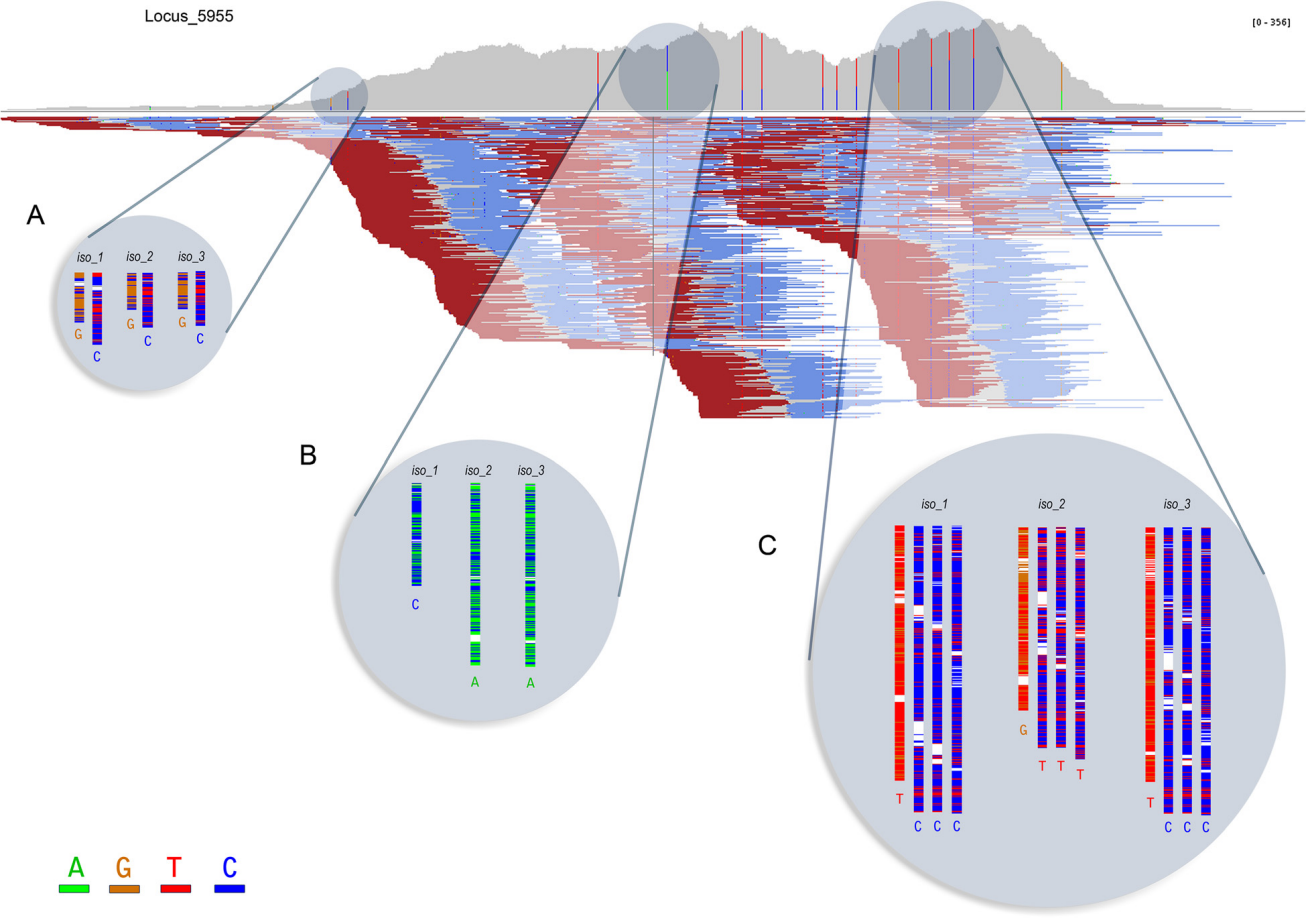
**Impact of read depth on the ability to discriminate between homeologs in polyploid genomes.** Read-depth schematic of *Locus_5955*, homologous to MPBQ-methyltransferase (VTE3). Vertical color bars inside plot represent the proportion of each putative distinct base (SNP) at a particular position **(A)** area with lower read coverage. **(B)** and **(C)** areas with higher coverage. Magnified areas show detail of the reads at each putative polymorphism site, for each one of the three transcript isoforms: iso_1 (*Locus_5955_1/3*), iso_2 (*Locus_5955_2/3*) and iso_3 (*Locus_5955_3/3*). Underneath each pile-up of reads appears the base called by Oases. The outlined graph represented in the figure corresponds to the second isoform of the locus (*Locus_5955_2/3*).

Read depth may have a significant impact on the ability to discriminate between homeologs in polyploid genomes. To examine this in detail, reads were mapped back to a particular chosen *Locus* (*Locus_5955*) and piled up to illustrate read depth (Figure 2). This *Locus* was chosen as an example because it is homologous to an enzyme in the tocol pathway, and was assembled into three isoforms, consistent with three oat homeologs. In effect, in areas with low-medium read-depth, differentiation between SNPs is problematic. For instance, the two zoomed-in putative SNPs in the low read-covered region of the assembled gene (Figure 2A) were overlooked by the assembly software and called as a single base for all three isoforms. However, the presence of a polymorphism is suggested by the number of reads bearing a different base. The expected high degree of homozygosity of the oat genotype used for RNA-Seq suggests that the single nucleotide variations observed in the assembled transcripts are most likely due to true homeoalleles and not to genetic heterozygosity. While, the latter cannot be excluded as source of SNPs, it is presumed to be less frequent than homeoallele sequence variation. In areas with more read depth (Figures 2B and C), SNP discrimination occurred. Generally, we observed effective discrimination by the assembler above a read depth of 75–100.

**Figure 3:**
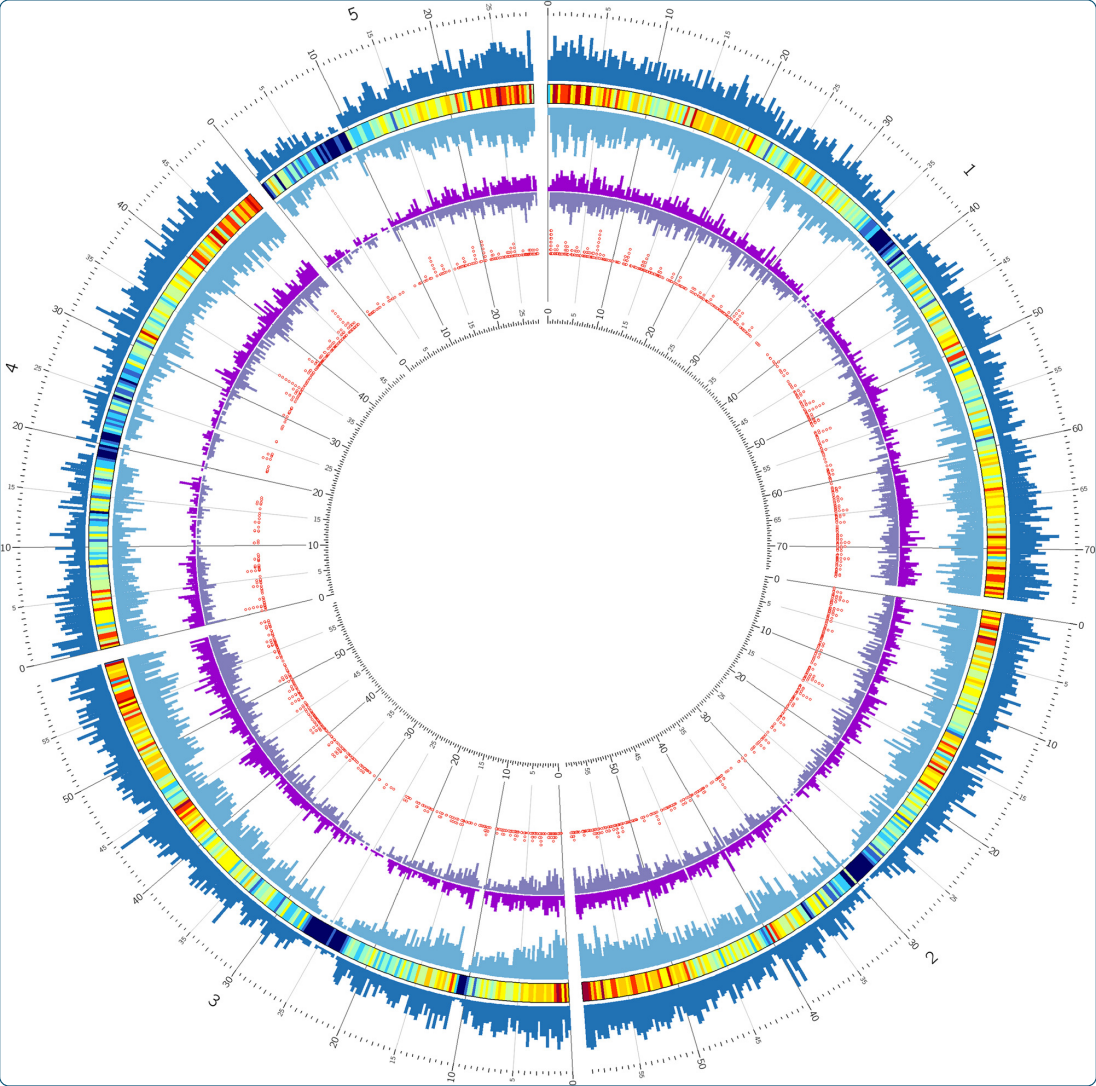
**Abundance and distribution of the *dn*OST transcript assembly compared to the Brachypodium genome.** SSR-bearing oat transcripts were distributed throughout the Brachypodium genome. External track shows Brachypodium gene density in both + (outside) and - (inside) strands in blue. In between both is the heat map of overall gene density. The middle track shows the density of the hits of oat alignments to Brachypodium, in both + (outside) and - (inside) strands (purple). Both Brachypodium and oat tracks are represented at the same scale. Inner-most track shows where the oat SSR-bearing transcripts align to Brachypodium genome sequence (red circles). Window size is 250 kb.

All *de novo* assembled *dn*OST transcripts were annotated (blastx, 1E-10, first hit) against GenBank’s non-redundant (NR) protein database (Additional file 6). Predicted proteins, including many with putative functions assigned, could be retrieved for 43,944 (82.4%) of the 53,339 *dn*OST transcript isoforms, which is a percentage similar or above previous studies involving plant species without sequenced genomes [10,14]. The redundancy in *dn*OST is indicated by the fact that just 13,362 of the annotated *dn*OST transcripts corresponded to unique NR peptides. Similarly, 10,133 Brachypodium predicted peptides were identified (blastx, 1E-10, HSF > 50%) that were similar to one or more *dn*OST transcript isoforms, with 31.5% of *dn*OST transcripts having two or more hits. Therefore, the transcripts identified over four oat seed developmental stages share homology to nearly 40% of the predicted 25,532 Brachypodium protein-coding genes [7]. The *dn*OST transcript sequences were uniquely anchored to the single best hit in the Brachypodium genome (Figure 3), and were found to be homogenously distributed in the Brachypodium genome and correlated (R = 0.88) with the Brachypodium coding sequence (cds) density, indicating that a broad diversity of genes is represented in *dn*OST.

### Functional classification of *dn*OST

**Figure 4:**
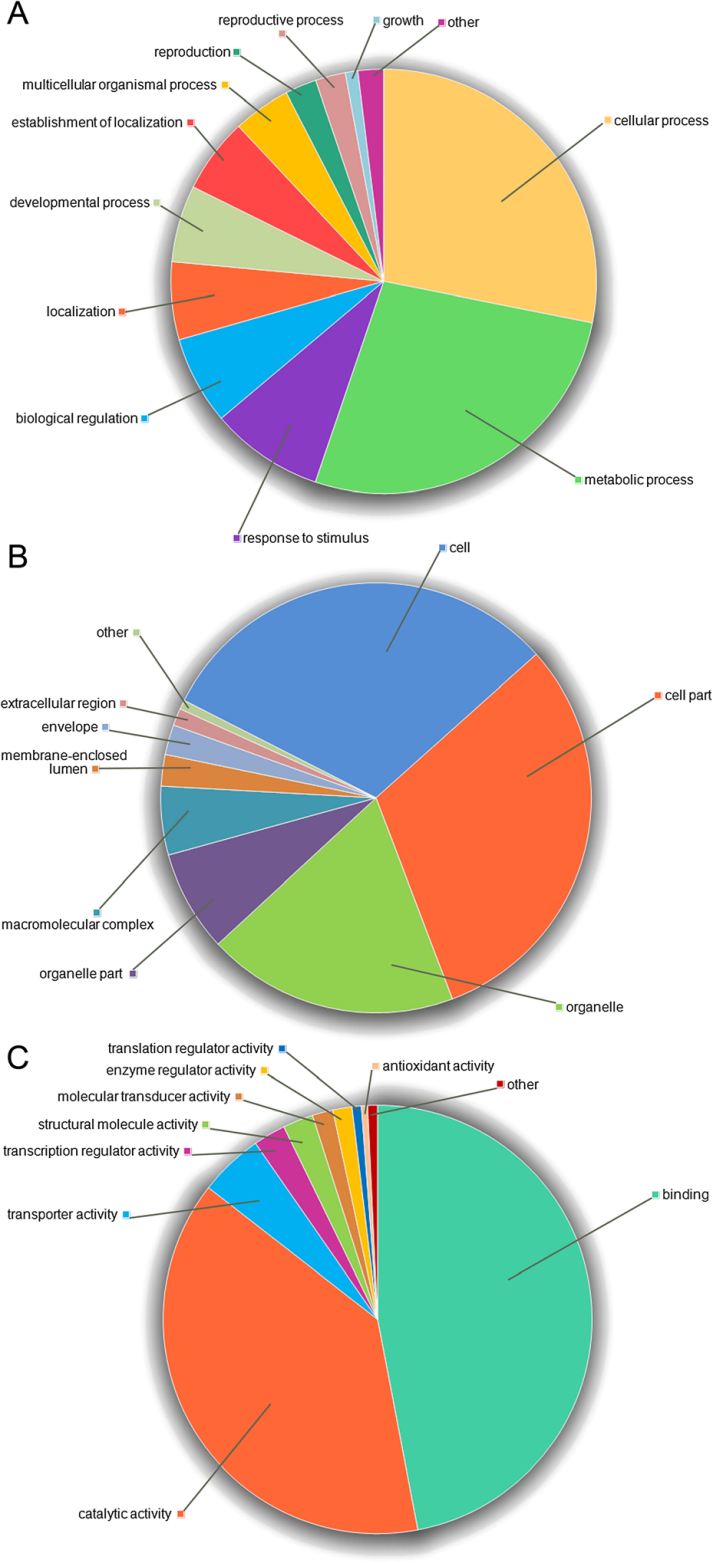
**Gene ontology analysis of the *dn*OST sequences.** Pie diagrams show the percentage distribution of each GO class. **(A)** Biological process **(B)** Cellular component and **(C)** Molecular function.

Functional classification of *dn*OST sequences was performed through a gene ontology (GO) categorization (Figure 4). The original 53,339 *dn*OST transcript isoforms were first clustered within and across *Loci* (see Methods) to 27,972 representative sequences, to reduce the redundancy of the original assembly. These clusters were queried (blastx, 1E-10, first hit) against Uniprot-Plants database, and subsequently annotated using the GO-Uniprot association file. An ontology annotation was found for 23,668 of the sequences (84.6%), of which 12,241 corresponded to unique proteins. The inferred GO terms were distributed in the three main GO domains as follows: biological process (8,064), cellular component (6,910), and molecular function (9,827). Biological process was mainly represented by cellular and metabolic processes (Figure 4A), representing more than 55% of the annotations, followed by response to stimulus (8.6%) and biological regulation (6.7%). When the sequences were categorized according to the cellular component main term (Figure 4B), 61.7% of them corresponded to cell or cell part categories, and 26.5% to organelle or organelle part. A hypergeometric statistical test was employed to identify over-represented (p < 0.05) GO categories and genes present in *dn*OST more often than expected by chance, as compared to Uniprot-Plants database (Additional file 7). Among over-represented cellular components were cell wall and other categories associated to developing tissues. Overrepresented molecular functions were: transferase activity, catalytic activity, nucleotide binding, ATP binding, kinase activity, phosphotransferase activity, and protein binding. Overrepresented biological processes were glycolysis and several other metabolic processes. Transcripts associated with the synthesis of important health-promoting compounds appear to be well-represented. For instance, the cell wall subcategory contained 339 unique tran-scripts, including peptidoglycan-based, cellulose- and pectin-containing, and chitin- and β-glucan-containing cell wall. The most represented GO subcategories within molecular function main term were binding (47%), catalytic activity (38.5%), transporter activity (4.8%), and transcription regulation activity (2.5%) (Figure 4C). All four molecular functions are involved in biosynthetic processes, reflecting the developing nature of the tissues analyzed.

**Figure 5:**
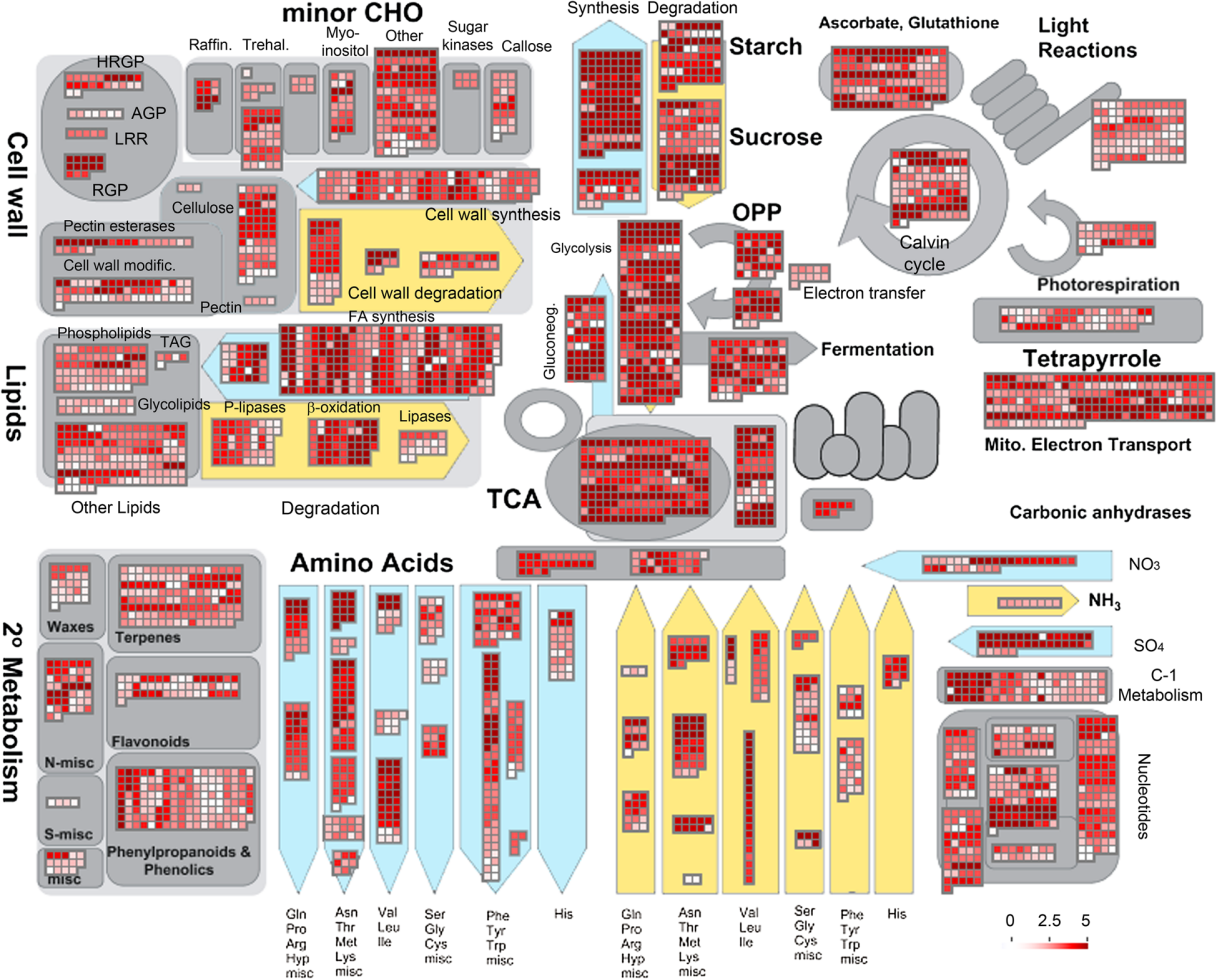
**MapMan overview of cellular metabolism.** Individual assembled transcripts are represented by colored squares. Color code scale is shown at the bottom right corner, and is based on the log2 of the RPKM values of each transcript (see materials and methods). Thus, more intense red refers to higher transcript abundance. Colored background figures represent different metabolic process; those highlighted in blue are biosynthetic pathways and those in yellow for degradation pathways.

In addition, all *dn*OST transcript isoforms were classified according to their major MapMan envisaged metabolic routes (Figure 5) and their normalized raw digital expression counts (Additional file 8) (see Methods for details). Results reinforce previous observations that the genes represented in *dn*OST are diverse in nature and cover many key cell processes. In agreement with GO classification, the MapMan metabolism overview functional classification also shows high numbers of transcripts involved in synthetic processes as compared to degradation processes, consistent with a developing tissue.

### Microsatellite markers in *dn*OST

Microsatellite (SSR) markers are broadly used for marker-assisted selection in crop breeding due to the ease of their implementation and co-dominant nature. We scanned the *dn*OST for gene-derived SSR markers with the potential to be used in oat breeding programs. In total, 4,639 SSRs were found within 4,128 different transcripts. A summary of the putative SSRs is shown in Additional file 9. Primers targeting the SSRs were designed when possible (Additional file 10). The most abundant SSRs were the tri-repeats (2841; 61.2%). The rest were distributed as follows: mono-repeats (1,144; 24.7%), di (455; 9.8%), tetra (126; 2.7%), penta (18; 0.4%), and hexa (55; 1.2%). Excluding mono-repeats, the percentage of di, tri, tetra, penta and hexa was 13.0, 81.3, 3.6, 0.5 and 1.6%, respectively. The SSR-bearing transcripts were aligned (blastx, 1E-10, best hit) against the Brachypodium genome sequence to pinpoint the syntenic location in which polymorphisms occur (inner-most track of Figure 3). Their distribution along the Brachypodium genome is uniform and consistent with gene density.

### Construction of oat seed gene indices *OatSeedRef100* and *OatSeedRef90*

To develop a comprehensive compendium of available oat seed expressed sequences, we combined the *dn*OST assembly with oat sequences published by other sources. The sequence information retrieved from the GenBank (as of Feb 2012) consisted of 17,711 oat seed EST sequences totaling 9,395,591 nt with an average length of 530.5 nt (min 50, max 846). These were concatenated with the 53,339 *dn*OST transcripts to build an index of 71,050 oat seed expressed sequences. We named this reference index *OatSeedRef100 v*1.0 (Additional file 11). Additionally, to reduce redundancy in *OatSeedRef100*, *OatSeedRef90* was created (Additional file 12) in a manner similar to the UniProt Reference Clusters databases UniRef100 and UniRef90 (http://www.uniprot.org/). Clustering reduces the presence of redundant sequences and base miscall errors, but can also eliminate highly similar homeoalleles. The *OatSeedRef90* non-redundant index is composed of 31,935 sequences, of which 14,805 are tentative consensus (clusters of two or more) and 17,130 singletons, for a total of 65,040,619 nt of sequence information. Of the 31,935 sequences, 23,016 (72.1%) were unique to the *dn*OST assembly, 6,521 (20.4%) were unique to the GenBank ESTs, and 2,398 (7.5%) resulted from merging of at least one GenBank sequence and one of our *de novo* transcript assemblies. Sequence lengths in *OatSeedRef90* range between 50 and 12,827 nt, with an average of 767.4 nt. Since the minimum contig length in *dn*OST was set to 100 nt, all of the shortest sequences (50–99 nt) derive from ESTs retrieved from GenBank. Clustering revealed that more than 63% of the oat ESTs in GenBank are overlapping or overlapped by sequences present in *dn*OST. The *OatSeedRef90* sequences were compared (blastx, 1E-10) with the Brachypodium and UniProt-Plants protein databases and 22,424 (70.2%) and 22,858 (71.6%) hits were retrieved, respectively. Only 5.3% of the unique Brachypodium proteins corresponded to the set of oat ESTs in GenBank, which indicates that the vast majority of the unique-protein targets come from our *de novo* assembly. This comparison further demonstrates that our RNA-Seq assemblies are an important source of novel oat cDNA sequences.

### Oat health-promoting compounds as a practical demonstration of *dn*OST utility

To demonstrate the utility of *dn*OST, we studied biosynthetic genes for three important health-promoting compounds in oats: avenanthramides, tocols, and β-glucans. Sequences of genes in the respective pathways from close relatives were downloaded from GenBank and other sources (Additional files 13 and 14) and the *dn*OST assembly was searched (blastn, 1E-10, HSP-id 80%) for homologous sequences. Homologous transcripts were found for all genes investigated (Additional file 13), which supports both the accuracy and completeness of the *dn*OST assembly.

**Figure 6:**
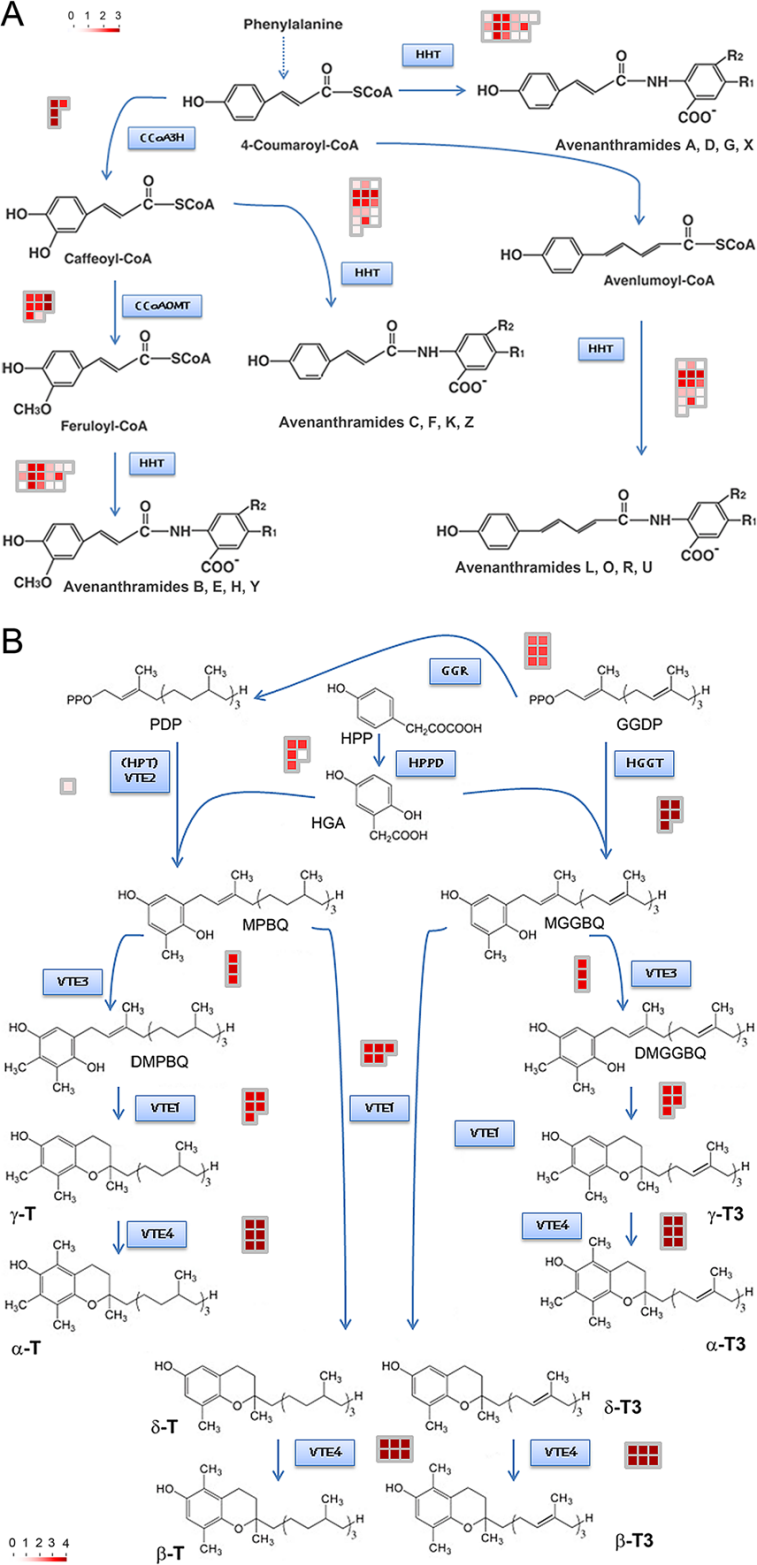
**Avenanthramide and tocol pathways showing homologous transcripts assembled in dnOST for each particular gene. (A)** Avenanthramides. Pathway adapted from [25]. **(B)** Tocols. Colored squares represent individual assembled transcripts. Color code scale is shown at the bottom and is based on the log2 of the RPKD values of each transcript (see materials and methods). CCoA3H: p-coumarate3-hydroxylase; HHT: hydroxycinnamoyl CoA:hydroxy-anthranilate N-hydroxycinnamoyl transferase; CCoAOMT: caffeoyl-CoA 3-O-methyltransferase; HPT: homogentisate phytyltransferase; GGR: geranylgeranyl diphosphate reductase; HPPD: 4-hydroxy-phenylpyruvate dioxygenase HGGT: homogentisate geranylgeranyl transferase; VTE1: 2-methyl-6-phytyl-1,4-benzoquinone cyclase; VTE3: 2-methyl-6-phytyl-1,4-benzoquinone/2-methyl-6-solanyl-1,4-benzo-quinone methyltransferase; VTE4: tocopherol methyltransferase; PDP: phytyl-diphosphate; GGDP: geranylgeranyl- diphosphate; HPP: p-hydroxyphenylpyruvic acid; HGA: homogentisic acid; MPBQ: 2-methyl-6-phytylbenzoquinol; DMPBQ: 2,3-dimethyl-6-phytyl-1,4-benzoquinone; MGGBQ: 2-methyl-6-geranylgeranylbenzoquinol; DMGGBQ: 2,3-dimethyl-5-geranylgeranylbenzoquinol; T: tocopherol; T3: tocotrienol.

The pivotal enzyme in the biosynthesis of avenanthramides is HHT, which catalyzes the final condensation reaction. Searches (blastn, 1E-50) against *dn*OST retrieved multiple homologous transcripts to the four reported oat HHT isoforms [GenBank:AB076980-83] (Additional file 14). Homologies were also found for genes coding two other key enzymes in avenanthramide synthesis, CCoAOMT and CCoA3H. For CCoAOMT, eight homologs were found in *dn*OST. Also, the complete CCoAOMT cds was obtained by expanding a previously existing GenBank sequence. The second enzyme, CCoA3H, is required for the synthesis of several of the most predominant avenanthramides. To our knowledge, no CCoA3H gene sequence has been reported for oats. We found four transcripts homologous to Brachypodium (90-91%) and barley (92%) CCoA3H genes. The expression of all avenanthramide synthesis homologs was quantified and is shown in heat maps for the main enzymatic steps in the pathway (Figure 6A). Homologous transcripts in the heat maps may represent homeoalleles or other highly similar sequences including paralogs. The results indicate that there are significant differences in the expression of homologous transcripts in many biosynthetic steps, suggesting that homeoalleles and/or paralogs may be differentially expressed.

Tocol accumulation profiles in developing oat seeds have only been studied recently, and distinctly different temporal patterns of accumulation for both tocotrienols and tocopherols were found [26]. Sequences of the genes in the tocol biosynthetic pathway were sought within the *dn*OST, and transcripts were found for all (Figure 6B). For instance, five transcripts exhibited homology to barley, Brachypodium, and wheat *HPPD* (Additional file 14). GGR catalyzes the reduction of GGDP to PDP, and six GGR homologues were assembled in *dn*OST which nearly cover the predicted complete cds. The committed step in the biosynthesis of tocopherols is condensation of HGA and PDP, catalyzed by HPT (VTE2), for which one oat transcript was found. Similarly, the committed step in tocotrienol biosynthesis is the condensation of HGA with GGDP, by HGGT. Five highly similar HGGT homologue transcripts were found in *dn*OST. Surprisingly, no HGGT homologue was found in the Brachypodium genome. Seeds of Brachypodium accumulate tocotrienols (unpublished data), thus it is likely that the HGGT homologue lies in an unsequenced region of the Brachypodium genome. The last three enzymes, VTE1, VTE3, and VTE4, are common for the synthesis of both tocopherols and tocotrienols, and homologues were found for all three genes. Thus, *dn*OST holds promise for linking gene expression to metabolic aspects of tocol accumulation in oat seeds.

β-glucans were the last health-promoting compounds evaluated. Cellulose-synthase (CES) and cellulose-synthase-like (CSL) sequences were downloaded from (http://cellwall.genomics.purdue.edu/) and compared to *dn*OST assembly. The retrieved matches included 36 CES and 24 CSL unique transcripts (Additional file 13). The average length of the transcript isoforms assembled for CSL and CES was 1,230 and 2,198, respectively. As an example, alignments of barley CLS-F6 (GenBank:EU267181) and the oat homologue (GenBank:GQ379900) with *dn*OST revealed homology to five isoforms (Additional file 14). Comparatively to barley, two insertions and one deletion at the 3′-end appears to produce an early stop codon. Similarly, ten assembled isoforms had 84-85% and 76-78% identity with their barley and Arabidopsis CES-A homologues, respectively.

## Discussion

Oat transcripts were *de novo* assembled from Illumina reads derived from four oat seed developmental stages. Assembly was performed with both Oases and Trinity, and the results were compared. Strict values were chosen for some parameters to assure a more precise assembly. To our knowledge this is the first comparison between Oases and Trinity in a polyploid organism, which makes our study a useful guide for future studies on transcriptome analysis in plants, where polyploidy is common. Several quality tests were performed to determine the robustness of each assembly; the length and number of assembled transcripts, and how precisely they match annotated databases are particularly valuable in this regard. In our assemblies we prioritized sequence accuracy over other criteria, such as total number of transcripts and length of total assembled sequence. This approach may risk losing rare transcripts, but overall it improves the quality of the assembly. Despite the fact that Trinity was specifically developed for transcript assemblies, our quality benchmarks indicated higher quality scores for Oases assemblies. It is likely that the reduced number of putative unique protein coding sequences from the Trinity assemblies is due in part to the shorter transcripts produced. Trinity developers initially tested its algorithms on diverse organisms such as fission yeast, mouse, and whitefly [24], but according to our results Trinity appears not to be as suitable for complex polyploid transcriptomes such as that of hexaploid oat. One of the reasons for this result may be that *k*-mer length is fixed at 25 nt; such a small number may not be sufficient to discriminate among highly similar homeologous or even paralogous sequences. Further, the short *k*-mer length causes Trinity to run considerably more slowly than Oases run with *67*-mer, since more words of size *k* per read have to be constructed and tested for alignment. If the multi-thread option of Trinity is not selected, Oases running with the fixed *25*-mer of Trinity finished 15–20 times faster than Trinity. Oases was shown to reconstruct a higher number of gene transcripts than Trinity on human and mouse datasets, although the accuracy of the assemblies was comparable [23]. Similarly, in the assembly of the tea plant (*Camellia sinensis*) transcriptome, Oases performed better than Trinity in most of the parameters studied [27]. However, Trinity produced better results for highly expressed genes. The authors also reported that Trinity run 20 times slower than Oases when the same *k-*mer value of 25 was used.

Based on our benchmark analysis, the Oases *67*-mer-assembly (*dn*OST) was selected as the representative for the seed oat transcriptome. The proportion of *dn*OST *Loci* with at least one homologue in other databases (UniRef, UniProt-Plants, and NR) ranged between 69.1 and 73.7% in these comparisons, and was highest for the UniProt-Plants database. These percentages increased up to 82.4% (UniProt-Plants, and NR) when all *dn*OST transcript isoforms were considered. Significantly, nearly 72% of the *Loci* (80.7% of the transcripts) in *dn*OST had a homologue within the collection of predicted Brachypodium peptides, which reinforces a previous study suggesting that Brachypodium can be an effective resource to assist oat genetics and genomics research [8,28]. Assembly errors and other sequence variation could produce fusion longer transcripts during the assembly. We do not believe that this occurred at a high level, as seen in the transcripts analyzed (Additional file 13). Although fusion proteins may not be completely discarded, that 31.5% of *dn*OST transcripts retrieved two or more Brachypodium predicted cds is more likely attributable to the presence of transcripts with more than one large conserved domain, or other regions with highly similar sequence (paralogs). Indeed, the Brachypodium genome shows six major chromosomal duplications covering 92.1% of the genome, which represent ancestral whole-genome duplications, and so detection of paralogs is likely to be a major factor in detecting more than one gene from blast searches [7].

For non-sequenced organisms, large-scale transcriptome assemblies from Illumina reads appear to be more robust than from Roche/454, presumably due to the higher coverage attained. In an assembly of chickpea transcripts obtained from Roche/454 RNA-Seq, only about 58% had similarity (blastx, 1E-10) to the Uniref50 database [13], vs. the nearly 70% in our assembly. In another study [20], Oliver *et al*. reported between 23,681 and 42,147 transcripts using high-throughput Roche/454 sequencing technology on four different oat genotypes, with average transcript lengths ranging between 561 and 598 nt, vs. the 53,339 transcripts averaging 1,043 nt in *dn*OST. Our transcriptome assembly appears to be accurate, as reflected by the results of blast searches against both plant and multi-organism databases. Thus *dn*OST greatly expands the current collection of oat expressed sequences. For instance, transcription factors are typically expressed at low levels and are most likely to be under-represented in EST databases; however, functional classification (GO) analysis of *dn*OST revealed a large group of putative transcription factor sequences.

We estimated that a 75–100 read depth is required for homeolog discrimination. To our knowledge this is the first study to provide such an estimate for a polyploid genome. In a *de novo* assembly of three individual human genomes, best false negative allele calls were obtained with considerably lower (20) read coverage [29]. Also, a 20× sequencing depth was required in the *de novo* assembly of an individual human genome with 75-nt read length to achieve a maximum contig size after which no further increase in length was observed [30]. Our assembly also suggests that homeologous genes in oat are both highly conserved and may remain functional, although confirming this would require extensive proteomic and enzymatic analyses. Moreover, the question of whether these sequences represent true homeologs, allelic variants, or artifacts is not empirically addressed in this study. Thus, caution may be advised when using *dn*OST for certain purposes. For instance, the assembly process may have merged multiple homeologs into a single sequence, shuffling SNPs among homeologs. This could be especially relevant where low sequence divergence prevented the assembler from resolving homeologs.

The homeologous forms in oat show similar or even a higher percentage of identity among them than was reported for wheat, with 90-99% identity at the nucleotide level and often identical at the amino acid level [9]. Polyploid species pose a challenge because of the presence of homeoalleles that may be difficult to deconvolute at the sequence level. To assemble highly similar homeologs separately requires a high number of reads and imposing strict parameters for assembly, such as larger *k*-mers, no mismatches allowed in *k*-mer alignments, and high minimum read coverage. Clearly, assembling with longer *k*-mers first requires longer reads. Currently, paired-end read sequencing using Illumina technology is limited to 150 nt, which still may be not sufficient to precisely discriminate highly similar homeologs in polyploid genomes. Conversely, stricter assembling parameters will reduce the number of transcripts. While assembling *dn*OST a balance between transcript redundancy and number was pursued, since obtaining a large number of transcripts was also a goal to make an oat library as complete and diverse as possible. Sequence redundancy is intrinsic to polyploid organisms, and this was proven to be the case in *dn*OST. Despite the continuous improvements in NGS technologies and assembly algorithms, it is still extremely challenging assembling highly homologous genes into separate isoforms.

There are numerous opportunities for practical use of *dn*OST. For example, the number of sequence polymorphisms that have been described in oat suitable to be used as molecular markers is very limited. For instance, only 106 SSR markers from oat were listed on the GrainGenes database as of November 2012 (http://www.graingenes.org). The more than 4,000 potential new genic SSR markers identified greatly expand the current SSR repository. While only a fraction of these may be found to be polymorphic between oat cultivars, validated polymorphic SSRs will provide a new resource for marker-assisted selection. As we found in oat, tri-nucleotide repeats are the most abundant (47-67%) in other plant species such as rice, corn, peanut, alfalfa, and Arabidopsis [10,11,14,31], indicative of the fact that they are probably preferentially present within transcripts to prevent frame shifts.

The first physically anchored hexaploid oat map has been recently completed [28]. A GoldenGate assay was employed for genotyping 3,072 SNPs as part of the mapping effort. To examine for the presence of those SNPs in the *dn*OST transcripts, searches (blastn, 1E-10) were conducted using the DNA sequence surrounding the SNP. This revealed that 2,160 (70.3%) of the SNPs in the GoldenGate assay were present in at least one transcript (Additional file 15). Because GoldenGate SNPs were first discovered using cDNA from different tissues, these results suggest *dn*OST to be a fairly comprehensive source sequences, despite its seed origin.

As a last example, transcripts for genes associated with the synthesis of health-promoting compounds are present in *dn*OST and thus novel information is available to examine molecular aspects of their synthesis. For instance, avenanthramides are unique to oat among cereal grains, but the genes involved in their synthesis have not been fully characterized. A key enzyme in avenanthramide pathway is HHT, from which there appear to be multiple isoforms that accept a wide range of substrates with different affinities [32]. Complete sequences for three oat HHTs (*AsHHT1-3*) have been reported [33], as well as a partial sequence for *AsHHT4* (GenBank:AB076980-83). We found twelve homologous transcripts to *AsHHT1*, eleven to *AsHHT2*, thirteen to *AsHHT3*, and thirteen *AsHHT4*, for a total of sixteen different transcripts. *Locus_17720_Transcript_1/2* (“*Locus_17720_1/2*”) had high sequence identity to *AsHHT1* (100% in cds and 98% ts). A second transcript isoform (*Locus_17720_2/2*) was the most similar (100% in both cds and ts) to *AsHHT2* (Additional file 15). These two *Loci* shared 96% identity with the differences located in the 3′UTR, suggesting that *AsHHT1* and *AsHHT2* are in fact homeologs. In another example, five *dn*OST transcripts had at least 95% homology in their predicted cds to *AsHHT4*: *Locus_14341* (99.3%), *Loci_15223_(1–2)/2* (95-96%), *Locus_12518* (96%), and *Locus_25525* (96%). By aligning all transcripts with the previously known partial *AsHTT4* cDNA we were able to extend the *AsHTT4* 244 bases towards the 5′ end to complete the cds. Thus, *dn*OST is useful not only to identify oat homologues for genes of interest, but also to obtain complete sequences of partially cloned oat genes.

## Conclusions

There are inherent challenges in developing an accurate oat gene transcript set because of its level of genome duplication, with post-processing of sequences often required to differentiate true homeologues from assembled artifacts. We have shown that our *de novo* transcript assembly of developing oat seeds obtained with Oases is able to differentiate highly similar genes to a significant extent. This indicates that the nearly 75 fold average coverage we obtained is deep enough to discern homoeoalleles and paralogs to some degree. Nevertheless, post-processing of sequences may still be required to establish whether these sequences represent true homeologs, particularly in transcripts with low coverage. Our study provides an optimized analytical pipeline for other researchers attempting to assemble transcriptome data from polyploid plant species. We validate that *dn*OST is an excellent source of diverse oat transcripts such as those associated with the synthesis of several oat seed compounds possessing health-promoting properties, and also served as a resource to identify several thousand new potential molecular markers. Thus the oat transcript assembly developed in this study will be useful for a variety of avenues of oat improvement.

## Methods

### Plant materials and growth conditions

Seeds of oat (*A. sativa* L.) genotype Ogle-C, derived from a single plant reselection with several rounds of selfing from the cultivar ‘Ogle’, were germinated in trays filled with potting mix in a growth chamber in short-day conditions (11 h light at 20°C, 13 h dark at 16°C) to promote vegetative growth. After 4 weeks, developing plants were transplanted to cones containing two parts soil-one part potting mix. Plants were grown to maturity in long-day photoperiod conditions (16/8 h light/dark) and 21°C day/16°C dark temperatures. Individual florets were tagged at the onset of anthesis, and developing de-hulled seeds were collected at 7, 14, 21, and 28 days after anthesis (daa). All samples were frozen in liquid nitrogen and stored in cryovials at −80°C until used for RNA extraction.

### RNA extraction, cDNA library construction and Illumina sequencing

Pools of approximately ten seeds at each developmental stage from each replicate tray were used for RNA extraction. Seeds were ground to a fine powder in a mortar and pestle with liquid nitrogen and RNA was extracted with the TRIzol® method (Invitrogen, Carlsbad, CA) following the manufacturer’s instructions. RNA was further purified with RNeasy plant columns (Qiagen, Valencia, CA) according to the standard protocol. After RNase-free DNase I (New England BioLabs, Ipswich, MA) digestion to eliminate DNA, quality and integrity of the RNA was determined with NanoDrop (Thermo Fisher Scientific Inc, Wilmington, DE) and RNA6000 Nano Assay on the Agilent 2100 Bioanalyzer™ (Agilent Technologies Inc, Santa Clara, CA), prior to cDNA library construction.

The Illumina TruSeq™ RNA Sample Preparation Guide was followed to prepare the samples for sequencing. RNA samples were quantified using Quant-iT™ RiboGreen® Assay (Invitrogen, Grand Island, NY) and then run on an Agilent Nano chip (Agilent Technologies Inc, Santa Clara, CA) to verify RNA integrity. Illumina library preparation, clustering and sequencing reagents were included in the Illumina TruSeq™ RNA library preparation kit, and used according to manufacturer’s recommendations (http://www.illumina.com). Subsequently, mRNA was purified by using poly-T oligo-attached magnetic beads and then fragmented and primed for cDNA synthesis. First strand was created using reverse transcriptase and random primers; the second strand was then synthesized to generate double-stranded cDNA. After a double SPRI purification, end-repairing and adenylation at the 3′-ends, adaptors were ligated to perform PCR enrichment. Multiplexed samples were pooled, 4 to a lane, and cut for a paired-end run using the Caliper XT (Caliper/Xenogen). Libraries were validated and quantified using a High Sensitivity Chip on the Agilent 2100 Bioanalyzer™, and PicoGreen Assay (Invitrogen) for KAPA qPCR (KAPA BioSystems), respectively. The Illumina cBOT was used for cluster generation following the manufacturer’s instructions, and the clustered flow cell was loaded onto the Illumina HiSeq 2000 machine. The samples were barcoded, multiplexed in 3 lanes, and sequenced using a paired-end read with 100 cycles. Initial base calling and quality filter of the Illumina HiSeq 2000 image data were performed by the default parameters of the Illumina HiSeq 2000 pipeline. Data was processed by the Illumina CASAVA v.1.8.0 software to generate fastq sequence files. The cDNA library preparation and sequencing reactions were conducted in the Biomedical Genomics Center, University of Minnesota.

This study is part of a broader project for which 12 libraries corresponding to 3 biological replications per developmental stage were individually tagged and sequenced. For this study, at each of four stages the largest library was used for analysis. A total of 145,004,260 Illumina 100-bp paired-end reads with average QS of 33 were generated as follows: 33,562,980 for 7-daa, 35,488,910 for 14-daa, 32,941,118 for 21-daa, and 43,011,182 for 28-daa. The raw reads were cleaned of primer adaptors, low quality reads, and reads with non-identified bases, to a total of 133,963,046 high-quality reads (average QS of 34.9), as follows: 31,240,158 for 7-daa, 33,015,532 for 14-daa, 30,432,964 for 21-daa, and 39,274,392 for 28-daa. Custom scripts were used to further trim these reads according their individual base-call QS, maintaining only the bases with a QS above 28.

### Short read de novo transcriptome assembly

For *de novo* assembly of the nearly 134 million Illumina short pair-ended reads two assembly packages were used. First, the Velvet (v.1.2.03)/Oases (v.0.1.22) algorithms [22,23] were run with the reads of all four stages combined and with different hash lengths (*k*-mers 51, 55, 59, 63, 67, 71, 75, 79, 83, 87, and 91) to optimize the assembly towards higher contiguity and specificity. The minimum number of times a *k*-mer has to be observed to be used in the assembly (coverage cutoff) was set to 10. Only 1 gap count (mismatch) per *k*-mer was allowed. Default levels were used for all other parameters. A different assembling strategy was used for the second package, Trinity (v. Nov-2011), due to the higher computational resources that its algorithms demand. It was not possible to assembly all four stages combined in the same run, as for Oases, and instead a separate assembly was performed for each one of the four stages. In the current version, Trinity allows only a 25 *k*-mer parameter. The minimum assembled transcript length was established at 100 nt for both assemblers. Since both packages use different nomenclature: ‘*sequence’* in Trinity, and ‘*transcript’* in Oases; for the sake of clarity the terms ‘transcript isoform’ or ‘isoform’ were used to refer to the final set of assembled sequences from either software packages. The terms ‘*Locus’* or ‘*Loci’* in italics were used to group together similar transcript isoforms, and corresponds to the concept of *loci* or *component* (*comp*) used by Oasis and Trinity, respectively, to call a cluster of contigs, as described in [23]. Merging of *OatSeedRef100* and oat seed GenBank EST sequences to obtain *OatSeedRef90* was performed with Cd-hit-EST [34] with a sequence identity threshold of 90%.

To benchmark the quality of the each assembly, two approaches were taken. First, commonly used quality parameters were measured: the median transcript length (N50), defined as the length of the longest sequence such that the sum of the lengths of sequences equal or longer is equal or greater to half the length of all assembled sequences, number of transcripts, and average transcript length. Second, a transcript isoform representative of each group of transcripts (*Locus*) was aligned (blastx) to three independent databases, using an e-value cutoff of 1e-10 and a minimum percentage of HSP identity (HSP-id) of 50%: the UniProt-Plants (UniProtKB), plant entries database release 2012_02 from http://ftp.uniprot.org/, consisting on the manually annotated Swiss-Prot and the automatically annotated TrEMBL, the UniRef50 database (http://www.uniprot.org/), and the putative gene coding sequences of the *Brachypodium distachyon* v7.0 genome annotation (http://www.phytozome.com/). Three transcript sequences were selected as representative of each *Locus*: i) the isoform with the higher confidence score (CS), regardless the length. CSs assigned by Oases are a heuristic measure that expresses the uniqueness of a transcript in a locus, ranging from 0 (low confidence) to 1 (high confidence). For Trinity assemblies, with no computed CS, the RPKM (Reads Per Kilobase of transcript model and per Million fragments mapped) expression value calculated by the assembly software was used as substitute for quality parameter; ii) the longest isoform with the higher CS; and iii) a representative transcript calculated by clustering all isoforms. Clustering was performed using Cd-Hit-Est with a sequence identity threshold of 95% and 90% alignment coverage for the shorter sequence.

### Gen Ontology (GO) functional descriptions and classification

The assembled transcript isoforms were first clustered (Cd-Hit-Est, id. 95%) and searched (blastx, 1E-10) against the Uniprot-tremble database. For the functional classification, matches were compared to the GO association Uniprot database downloaded from GOTreePlus (http://hcil.snu.ac.kr/research/gotreeplus), and their GO terms retrieved. GO functional categorization was visualized with GOTreePlus [35]. BiNGO [36] was used to perform hypergeometric statistical test of significance (p-value < 0.05) to assess GO term enrichment. BiNGO highlights GO terms found within a gene list more often than expected by chance. To adjust for multiple hypotheses testing, a Benjamini & Hochberg false rate discovery correction was performed to control the type I error rate [37].

Scrutiny of transcript diversity and abundance was performed with MapMan [38]. The barley (*Hordeum vulgare*) Hvu_Affy database of annotated terms (http://mapman.gabipd.org) was used as reference. Accordingly, oat transcripts were first compared (blastn, 1E-5, 50% id.) against the Hvu_Affy sequences downloaded from the Affymetrix® website (http://www.affymetrix.com). To quantify transcript abundance, raw gene expression counts were computed as the number of the original Illumina reads that mapped back to the *de novo* assembled transcript isoform sequences. Alignment of the pair-end reads was performed by means of Bowtie [39], with default parameters. Transcript expression counts were calculated as the number of unique reads which aligned to the transcript assemblies, and were normalized with the RPKM method (Reads Per Kilobase of transcript model per Million mapped reads) as described in [40], using the Bowtie output mapped counts and customized scripts. For color-coded representation (heat map), the log2 of the RPKM-normalized expression counts was used. For pile-up representation of reads in Figure 4, Bowtie aligned output reads were converted to bam format, sorted, and indexed using the SAM tools [41]. Reads were then visualized with the Integrative Genomics Viewer [42]. Alignment of transcripts in the cases studied was performed with the ClustalW algorithm in the MEGA v.5.05 tool package [43], using by default parameters.

### SSR detection

Simple sequence repeats (SSRs) were identified using MISA [44] and filtered to represent unique polymorphisms by customized scripts. The minimum number of nucleotide repeats required was 10 mono-nucleotide repeats, 7 for di-nucleotide, and 5 for other repeats, that is, tri-, tetra-, penta-, and hexanucleotide repeats, and the maximum number of bases interrupting 2 SSRs in a compound SSR was set to 100 bp. Primers design was performed in batch with Primer3 [45], using default parameters and Perl scripts. For the Circos [46] representation, the SSR-harboring transcript sequences were compared (blastx, 1E-10, id 50%) against the predicted *Brachypodium distachyon* translated coding sequences, retrieving the first match. Genomic positions of the corresponding Brachypodium genes and exons, chromosome localization, as well as the strand in which each gene is located (+/−), were taken from the .gff3 file downloaded from (http://www.phytozome.com/) using a window size of 250 kb. The central position of each gene interval was taken as the location for that gene. Heat map of gene density was calculated using information of both strands and customized scripts.

## Abbreviations

β-glucan: (1 → 3) (1 → 4)-β-D-glucan; bp: Base pair; cds: Coding sequence; ts: Transcript sequence; daa: Days after anthesis; GO: Gene ontology; HSF: Homeologous set file; HSP-id: High-scoring pair identity; MAS: Marker assisted selection; nt: Nucleotide; RPKM: Reads Per kilobase of transcript model per million mapped reads; SPRI: Solid phase reversible immobilization; SSR: Simple sequence repeat; SNP: Single nucleotide polymorphism; CCoA3H: P-coumarate 3-hydroxylase; CCoAOMT: Caffeoyl-CoA 3-O-methyltransferase; DMGGBQ: 2,3-dimethyl-5-geranylgeranylbenzoquinol; DMPBQ: 2,3-dimethyl-6-phytyl-1,4-benzoquinone; GGDP: Geranylgeranyl-diphosphate; GGR: Geranylgeranyl diphosphate reductase; HGA: Homogentisic acid; HPP: P-hydroxyphenylpyruvic acid; HHT: Hydroxycinnamoyl CoA:hydroxyanthranilate N-hydroxycinnamoyl transferase; HPT: Homogentisate phytyltransferase; HPPD: 4-hydroxyphenylpyruvate dioxygenase; HGGT: Homogentisate geranylgeranyl transferase; MGGBQ: 2-methyl-6-geranylgeranylbenzoquinol; MPBQ: 2-methyl-6-phytylbenzoquinol; PDP: Phytyl-diphosphate; VTE1: 2-methyl-6-phytyl-1,4-benzoquinone cyclase; VTE3: 2-methyl-6-phytyl-1,4-benzoquinone/2-methyl-6-solanyl-1,4-benzoquinone methyltransferase; VTE4: Tocopherol methyltransferase; T: Tocopherol; T3: Tocotrienol.

## Competing interests

The authors declare that they have no competing interests.

## Authors’ contributions

DFG and JJGG conceived and designed the study. JJGG and ZJT performed bioinformatics analysis. JJGG performed data analysis and developed graphics. JJGG and DFG wrote the manuscript. All authors read and approved the final manuscript.

## Supplementary Material

Additional file 1 Excel spreadsheet containing statistics of the quality parameters used to asses the performance of Oases and Trinity assemblies. Click here for file

Additional file 2 **Graphs containing representations of several quallity parameters for the Oases and Trinity assemblies.** Comparison (blastx) of the assemblies against well-validated databases: (A, D) the complete set of translated gene coding sequences of *Brachypodium distachyon* (B, E) UniRef50 database (C, F) UniProt-Plants database. Click here for file

Additional file 3 **A fasta file containing the total 53,339 sequences of the *dn*OST assembly.** Sequences were *de novo* generated with Velvet/Oases using a k-mer 67 by assembling nearly 134 million quality-filtered 100-bp paired-end Illumina reads. Click here for file

Additional file 4 **Graph displaying the frequency distribution of the *de novo *assembled transcript lengths.** Assembly was performed with Velvet/Oases and k-mer of 67 nt (*dn*OSt). Click here for file

Additional file 5 **File containing the Homeologous Set File (HSF).** It has 22,818 families of homeologous relationships and close paralogs. It can be viewed as an Excel spreadsheet or with any word processor. Click here for file

Additional file 6 **File containing *de novo *assembled *dn*OST transcripts annotation (blastx, 1E-10, first hit) against GenBank’s non-redundant (NR) protein database.** Putative functions could be assigned for 43,944 transcripts. It can be viewed as an Excel spreadsheet or with any word processor. Click here for file

Additional file 7 Excel spreadsheet containing the over-represented GO categories and genes. Click here for file

Additional file 8 **File containing the normalized raw digital expression counts of *dn*OST transcripts, normalized with the RPKM method (Reads Per Kilobase of transcript model per Million mapped reads) as described in [**[40]**].** It can be viewed as an Excel spreadsheet or with any word processor. Click here for file

Additional file 9 **File containing the *dn*OST gene-derived SSR markers with the potential to be used in oat breeding programs.** In total, 4,639 SSRs were found within 4,128 different transcripts. It is better viewed using a word processor. Click here for file

Additional file 10 **Primers targeting the SSRs described in ** Additional file 10**.** It can be viewed as an Excel spreadsheet or using a word processor. Click here for file

Additional file 11 **A fasta file containing the *OatSeedRef100 v*1.0.** A comprehensive compendium of available oat seed expressed sequences, constructed by combining the *dn*OST assembly with oat sequences published by other sources (GenBank) to build an index of 71,050 oat seed expressed sequences. Click here for file

Additional file 12 **A fasta file containing the *OatSeedRef90*, created by clustering the sequences in *OatSeedRef100 v*1.0 to reduce redundancy.** Click here for file

Additional file 13 **Excel spreadsheet containing homologous *dn*OST transcripts to sequences (reciprocal matches) of genes in the pathways of oat healthy compounds (avenanthramides, tocols (vitamin E), and β-glucans) downloaded from close relatives.** Click here for file

Additional file 14 **Word files containing alignments of the *dn*OST homologous transcripts to key enzymes in avenanthramide, tocol, and β-glucan synthesis.** Click here for file

Additional file 15 **Text file containing the SNPs in the GoldenGate platform developed by [**[28]**] for which at least a match *dn*OST transcript has been found.** Click here for file
