# Supplementary material for: Analysis and annotation of the hexaploid oat seed transcriptome
Source: BMC Genomics. 2013 Jul 11;14:471. doi: 10.1186/1471-2164-14-471 (PMC3720263; doi:10.1186/1471-2164-14-471)
Supplement: Additional file 2 — Graphs containing representations of several quallity parameters for the Oases and Trinity assemblies. Comparison (blastx) of the assemblies against well-validated databases: (A, D) the complete set of translated gene coding sequences of Brachypodium distachyon (B, E) UniRef50 database (C, F) UniProt-Plants database. [file 1471-2164-14-471-S2.docx]

Quality measurements for Velvet/Oases assemblies

**Additional file 2** **(A)** blastx searches against the complete set of translated gene coding sequences of Brachypodium distachyon, using the longest transcript isoform, the isoform with highest confidence value and the transcript consensus (TC) for each of the Velvet/Oases assemblies. k-mers ranging 51-91 nt were used. Total number of contigs, scaffolds (transcripts) and number of unique groups of isoforms (unique isoforms), as well as the N50, average contig and average scaffold (transcript) length were also included to have an overall picture of the quality of each assembly.

**Additional file 2 (B)** blastx searches against the UniRef50 database, using the longest transcript isoform, the isoform with highest confidence value and the transcript consensus (TC) for each of the Velvet/Oases assemblies. k-mers ranging 51-91 nt were used. Total number of contigs, scaffolds (transcripts) and number of unique groups of isoforms (unique isoforms), as well as the N50, average contig and average scaffold (transcript) length were also included to have an overall picture of the quality of each assembly.

**Additional file 2 (C)** blastx searches against the UniProt Plants (UniProtKB) plant entries database, using the longest transcript isoform, the isoform with highest confidence value and the transcript consensus (TC) for each of the Velvet/Oases assemblies. k-mers ranging 51-91 nt were used. Total number of contigs, scaffolds (transcripts) and number of unique groups of isoforms (unique isoforms), as well as the N50, average contig and average scaffold (transcript) length were also included to have an overall picture of the quality of each assembly.

Quality measurements for Trinity assemblies

**Additional file 2 (D)** blastx searches against the complete set of translated gene coding sequences of *Brachypodium distachyon*, using the longest transcript isoform, the isoform with highest confidence value and the transcript consensus (TC) for each of the Trinity assemblies. An independent assembly was performed for each of the four seed stages (7-daa, 14-daa, 21-daa and 28-daa). Total number of scaffolds (transcripts) and number of unique groups of isoforms (unique isoforms) (scale on left y-axis), as well as the N50 and average scaffold (transcript) length (scale on right y-axis), were also included to have an overall picture of the quality of each assembly.

**Additional file 2 (E)** blastx searches against the complete set of translated gene coding sequences of UniRef50 database, using the longest transcript isoform, the isoform with highest confidence value and the transcript consensus (TC) for each of the Trinity assemblies. An independent assembly was performed for each of the four seed stages (7-daa, 14-daa, 21-daa and 28-daa). Total number of scaffolds (transcripts) and number of unique groups of isoforms (unique isoforms) (scale on left y-axis), as well as the N50 and average scaffold (transcript) length (scale on right y-axis), were also included to have an overall picture of the quality of each assembly.

**Additional file 2 (F)** blastx searches against the UniProt Plants (UniProtKB) plant entries database, using the longest transcript isoform, the isoform with highest confidence value and the transcript consensus (TC) for each of the Trinity assemblies. An independent assembly was performed for each of the four seed stages (7-daa, 14-daa, 21-daa and 28-daa). Total number of scaffolds (transcripts) and number of unique groups of isoforms (unique isoforms) (scale on left y-axis), as well as the N50 and average scaffold (transcript) length (scale on right y-axis), were also included to have an overall picture of the quality of each assembly.
