# Supplementary material for: Analysis and annotation of the hexaploid oat seed transcriptome
Source: BMC Genomics. 2013 Jul 11;14:471. doi: 10.1186/1471-2164-14-471 (PMC3720263; doi:10.1186/1471-2164-14-471)
Supplement: Additional file 4 — Graph displaying the frequency distribution of the de novo assembled transcript lengths. Assembly was performed with Velvet/Oases and k-mer of 67 nt (dnOSt). [file 1471-2164-14-471-S4.docx]

Length (nt)

**Figure S2** Frequency distribution of the *de novo* assembled transcript lengths. Assembly was performed with Velvet/Oases and k-mer of 67 nt (*dn*OSt).
