# Supplementary material for: Analysis and annotation of the hexaploid oat seed transcriptome
Source: BMC Genomics. 2013 Jul 11;14:471. doi: 10.1186/1471-2164-14-471 (PMC3720263; doi:10.1186/1471-2164-14-471)
Supplement: Additional file 14 — Word files containing alignments of the dnOST homologous transcripts to key enzymes in avenanthramide, tocol, and β-glucan synthesis. [file 1471-2164-14-471-S14.docx]

Dataset S11

Avenanthramide synthesizing genes

1. HHT1-3

1. HHT4

1. CCoAOMT

1,2,5

1,2,5

3,4

2

1

5

1,2,3,4,5

1. CCoA3H

Tocopherol and tocotrienol synthesizing genes

1. HPPD

1. HPT (VTE2)

1. GGR

1. HGGT

1. VTE1

1. VTE3

1. VTE4

β-glucan synthesizing genes

1. CSL

1. CES
